# Therapeutic Intervention in Cancer by Isoliquiritigenin from Licorice: A Natural Antioxidant and Redox Regulator

**DOI:** 10.3390/antiox11071349

**Published:** 2022-07-11

**Authors:** Zhu Zhang, Ken Kin-Lam Yung, Joshua Ka-Shun Ko

**Affiliations:** 1 Teaching and Research Division, School of Chinese Medicine, Hong Kong Baptist University, Hong Kong, China; zhangzhu@hkbu.edu.hk; 2 Department of Biology, Hong Kong Baptist University, Hong Kong, China; 3 Golden Meditech Centre for Neuroregeneration Sciences, Hong Kong Baptist University, Hong Kong, China; 4 Centre for Cancer and Inflammation Research, School of Chinese Medicine, Hong Kong Baptist University, Hong Kong, China

**Keywords:** isoliquiritigenin, natural antioxidant, autophagy, redox regulation, reactive oxygen species

## Abstract

Oxidative stress could lead to a variety of body dysfunctions, including neurodegeneration and cancer, which are closely associated with intracellular signal transducers such as reactive oxygen species (ROS). It has been suggested that ROS is the upstream regulator of autophagy, and that it provides a negative feedback regulation to remove oxidative damage. Defects in the ROS-autophagic redox homeostasis could lead to the increased production of ROS and the accumulation of damaged organelles that in turn promote metabolic reprogramming and induce tumorigenesis. One significant characteristic of pancreatic cancer is the reprogramming of cellular energy metabolism, which facilitates the rapid growth, invasiveness, and the survival of cancer cells. Thus, the rectification of metabolic dysfunction is essential in therapeutic cancer targeting. Isoliquiritigenin (ISL) is a chalcone obtained from the plant *Glycyrrhiza glabra*, which is a powdered root licorice that has been consumed for centuries in different regions of the world. ISL is known to be a natural antioxidant that possesses diversified functions, including redox regulation in cells. This review contains discussions on the herbal source, biological properties, and anticancer potential of ISL. This is the first time that the anticancer activities of ISL in pancreatic cancer has been elucidated, with a coverage of the involvement of antioxidation, metabolic redox regulation, and autophagy in pancreatic cancer development. Furthermore, some remarks on related compounds of the isoflavonoid biosynthetic pathway of ISL will also be discussed.

## 1. Introduction

Pancreatic cancer is a lethal malignancy predominated by its most common type, pancreatic ductal adenocarcinoma (PDAC), which accounts for more than 90% of all malignant pancreatic carcinomas in the world. PDAC is characterized by late diagnosis, poor prognosis, and early metastasis, with limited treatment options and an unsatisfactory response to chemotherapy, partly due to its progressive nature and high level of chemoresistance. Surgical resection is considered to be the treatment that may provide the potential cure for pancreatic cancer, whereas chemotherapy remains the only hope for non-resectable and metastatic cases. Unfortunately, chemotherapy is often associated with many drawbacks, including chemoresistance and serious systemic side effects [1]. Thus, signal transduction target therapy, immunotherapy, stem-cell therapy, modulation of the stroma, and inhibition of cancer metabolism have been emerging for the treatment of PDAC [2,3]. Among different tumorigenic mechanisms, metabolic dysregulation has largely contributed to the formation and progression of pancreatic cancer, and it offers new insights into the development of novel adjuvants for the modulation of the associated mechanism.

Barriers formed by the dense fibrotic stroma hinder pancreatic cancer from obtaining sufficient nutrients and oxygen [4]. Similar to other malignancies, pancreatic cancer has enormous energy demands by shifting the TCA cycle to aerobic glycolysis, a process known as the “Warburg effect” [5]. Unlike normal cells, mitochondrial oxidative phosphorylation is not favored by pancreatic cancer cells. As cancer cells usually create a hypoxic environment, ATP and lactate will be generated. Multiple pathways are involved in altering the glucose metabolism of PDAC. An evaluation of the effects of any possible anticancer chemicals/herbs/drugs by targeting a single-pathway mechanism in pancreatic cancer may not be feasible. Thus, a study on the correlation between metabolic regulation and multiple pathways and genes would become necessary [6].

The role of antioxidants is to detoxify and block the formation of reactive oxygen species (ROS), which are detrimental to human cells and induce DNA damage. The latter is a characteristic of cancer cells to promote the formation and progression of tumors. Thus, it is a general idea that the consumption of antioxidants in food and dietary supplements could effectively prevent or alleviate cancer development, including that in the pancreas [7,8]. It has been reported in an Italian case-control study that a diet high in “dietary total antioxidant capacity” is inversely associated with pancreatic cancer risk [9]. It is, however, interesting to note that cancer cells also possess an inherent mechanism that reduces ROS through its own antioxidant program, which can facilitate tumor growth by conferring a more reduced intracellular system, for instance, in order to maintain its resistance against apoptosis [10]. Hence, the modulation of the redox state to attenuate such a “ROS-detoxification program” can contribute to the reduction of tumorigenesis, which confers a potential target for cancer therapy [11]. 

Many natural flavonoids are known to be powerful antioxidants that possess a variety of bioactivities. Nevertheless, there is increasing evidence that among these diversified cellular functions, the role played by natural flavonoids may involve redox regulation in cells independent of their antioxidant properties [12]. The antioxidant defense system of the human body aims to reduce the level of harmful ROS in order to conserve cell integrity while maintaining enough ROS for essential body processes such as cell signaling and redox regulation. Antioxidants can help to scavenge excessive ROS production in the body and alleviate oxidative stress, but this could sometimes lead to adverse effects in the body, including mortality. Such a phenomenon that results from the severe disturbance of antioxidative activity and ROS balance in the body has been given the name “antioxidative stress” [13]. Isoliquiritigenin (ISL) is a chalcone-type flavonoid derived from licorice compounds, which can be found in food, beverages, and tobacco products. It has been proposed as a natural antioxidant that manifests cardioprotection as well as one that has acquired the potential to balance the cellular redox status. The latter property of ISL is believed to be contributed by its ability to directly trigger the AMP-activated protein kinase (AMPK) signaling pathway that modulates glucose homeostasis to protect against hypoxia-induced cardiomyocytes injury [14]. ISL could attenuate oxidative stress partly through the mediation of ROS [15]. Reports have suggested that the beneficial role of ISL in cardioprotection through the alleviation of myocardial oxidative stress may also involve the activation of the nuclear factor E2-related factor 2 (Nrf2)/heme oxygenase 1 (HO-1) signaling [16]. In addition, ISL has also demonstrated organoprotection against hepatotoxicity and acute pancreatitis through a similar mode of action via the inhibition of oxidative stress and the modulation of the Nrf2/HO-1 pathway [17,18]. 

## 2. Licorice (Glycyrrhiza Radix)

Licorice is the powdered dried root or rhizome of the plant *Glycyrrhiza Radix*, which is named “*Gan Cao*” in Chinese (Table 1). It belongs to a member of the legume (pea) family, which has been extensively used in the daily life of people in both the Orient and the West since ancient times. The earliest records of its medical use can be dated back to the era of the ancient Assyrians, Egyptians, Chinese, and Indians [19], while the Greeks were the first to make therapeutic use of licorice in Europe. There are about 20 species of the plant under the genus *Glycyrrhiza*, which is native to Europe, Asia, North and South America as well as Australia. The main source of licorice is *Glycyrrhiza glabra* L. in Europe and *Glycyrrhiza uralensis* Fisch. in China. It is remarkable as it is the “sweet root”, a natural sweetener that is more than 50 times as sweet as sucrose and which has profound pharmaceutical activity. *Glycyrrhiza Radix* can be found in many regions in Europe and Asia [20]. The key ingredient in the root that provides its sweetness is glycyrrhizin. The plant also contains various sugars, starch, flavonoids, saponins, sterols, amino acids, gums, and essential oil [21]. The high stability of licorice under different extraction forms and its long-lasting natural sweetness allow it to be used in various applications in people’s daily lives [22]. During the 18th century in England, licorice extracts began to be used as food sweeteners in candies and snacks, while the aroma of licorice after processing, which bears a unique tangy flavor, has made it a key ingredient in American tobacco [23]. 

The most common medicinal use of licorice is to treat upper respiratory diseases such as asthma, chronic cough, sore throat, and bronchitis. A well-known folk use of licorice in Europe is in the treatment of gastric and intestinal ulcers by lowering the acid level and coating the stomach wall with a protective gel. In addition, it can also help in relieving pain from toothaches by chewing the root. Sometimes, licorice can be used as poultice, which is effective in treating dermatitis and skin infections [22,23]. In ancient China, the applications of *Glycyrrhizae Radix* have been recorded in *Shen Nong Ben Cao Jing*, the earliest and most authoritative Chinese herbal pharmacopeia, written in 200 BC. It is one of the most widely and commonly used herbs in many Traditional Chinese Medicine (TCM) formulations. It can be found in the Chinese provinces of Gansu and Inner Mongolia as well as Shaanxi, Shanxi, Liaoning, Jilin, Heilongjiang, Hebei, Qinghei, and Xinjinag, with many growing it under Good Agricultural Practice standards. It is common to use *Glycyrrhizae Radix* to balance the effects of TCM prescriptions that contain multiple herbal ingredients [24].

Licorice is composed of more than 20 triterpenoids and nearly 300 flavonoids, with the key active constituents being glycyrrhizin, glycyrrhetinic acid, licochalcone A, licochalcone E, glabridin, and liquiritigenin [25,26] (Table 2). The licorice triterpenoid glycyrrhizin and its derivatives have been studied for their potential oncopreventive and oncotherapeutic functions [27]. On the contrary, it is also known that the chronic use of licorice may induce nephrotoxicity, which causes hypertension by inducing a hyper-mineralocorticoid state to suppress the renin-angiotensin system, possibly due to its glycyrrhetinic acid content [28]. The four known biomarker components of *Glycyrrhizae Radix* are glycyrrhizin, ISL, liquiritigenin, and liquiritin. Among the four, glycyrrhizin exhibits the highest plasma concentration and the longest half-life following the oral administration of the *Glycyrrhizae Radix* extract, while plasma concentrations of ISL and liquiritigenin would be restored to initial concentrations after 4–10 h of extract consumption due to metabolic conversion from other major flavonoids [29].

## 3. Isoliquiritigenin (ISL) and Its Biological Properties

In the past, research on licorice was focused on glycyrrhizin, but recently, other bioactive constituents have also been vigorously studied for different therapeutic purposes, including their potential neuroprotective and anticancer effects. Among these is ISL, a simple chalcone derivative of licorice, with the molecular formula of C_15_H_12_O_4_ and a molecular weight of 256.26. Its IUPAC name is (E)-1-(2,4-dihydroxyphenyl)-3-(4-hydroxyphenyl)prop-2-en-1-one [30]. This isoflavonoid has been found to possess a broad range of pharmacological properties, including anti-inflammatory, anti-viral, anti-microbial, anti-oxidative, immunomodulatory, hepatoprotective, and cardioprotective actions [31]. 

We have recently performed a network pharmacology study to assess all the potential targets that are common between PDAC and *Glycyrrhizae Radix*. Based on the 3961 well-established PDAC-related genes collected from the web-available Therapeutic Target Database, DisGeNET, and OMIM, and on the 2211 disease-conditioning genes of *Glycyrrhizae Radix* collected from the TCMID and NPASS databases, a PPI network (the Protein–Protein Interactions Network) was established by String Database to visualize their interrelationship in order to identify 95 filtered, potential, common gene targets. From the heatmap generated, multiple gene targets in the treatment of pancreatic cancer by major *Glycyrrhizae Radix* isoflavonoid constituents, including ISL, calycosin, and formononetin, have been identified. Among these, ISL possesses the most oncogenic targets. 

### 3.1. Anti-Inflammatory Effects of ISL

ISL is recognized for its anti-inflammatory effect. It has been proven to suppress the vascular cell adhesion molecule (VCAM-1) expression and mRNA accumulation of E-selectin on activated human umbilical vein endothelial cells (HUVEC), which play an important part in inflammation. Moreover, ISL could downregulate the cell adhesion molecule proteins in TNF-α-activated cells by blocking the nuclear translocation of NF-κB and IκBα degradation [32]. Furthermore, ISL has produced anti-inflammatory effects via anti-nephritic action [33] and the regulation of macrophages [34]. Additionally, ISL could inhibit the production of IL-6 and IL-12 p40 [35]. ISL has also demonstrated its inhibitory effects on both memory Th2 and antigen-induced Th2 inflammation by suppressing the production of IL-4 and IL-5; thus, it could serve as an anti-asthmatic agent [36].

### 3.2. Anti-Microbial Activity of ISL

ISL has shown a wide spectrum of anti-bacterial activities towards both Gram-positive and Gram-negative bacteria. ISL has been shown to inhibit the growth of the Gram-positive *Mycobacterium bovis* and reduce the putative dehydratase enzyme via fatty acid synthase II in *Mycobacterium tuberculosis* [37]. ISL has been reported to strongly suppress the growth of the Gram-negative bacteria *Ralstonia solanacearum*, *Fusobacterium nucleatum*, *Porphyromonas gingivalus*, and *Prevotella intermedia* [38,39]. ISL has also exhibited the best minimum bacterial concentration, an index being used to determine antimicrobial activity, ranging from 31.2 to 62.5 μg/mL against *S. aureus* and *S. mutans* [40]. ISL also possesses anti-viral capacities against the influenza virus and the hepatitis C virus (HCV). The inhibition of viral replication with an effective concentration of 50% (EC50) is 24.7 μM [41]. Likewise, anti-HCV activity with an inhibitory concentration of 50% (IC50) is 3.7 μg/mL [42].

### 3.3. Anti-Diabetic Effects of ISL

ISL has been served as an inhibitor of aldose reductase. Aldose reductase plays a major role in developing diabetic angiopathy. The structure of a γ,γ-dimethylchromene ring in ISL is partly responsible for the inhibitory effects of aldose reductase, which prevents osmotic stress during hyperglycemia [43]. Meanwhile, ISL could attenuate the symptoms associated with a high glucose (HG) level via the suppression of HG-induced mesangial fibrosis. ISL has also been shown to inhibit the transforming growth factor (TGF)-β receptor I and II kinase by attenuating their downstream Smad signal transduction and decreasing the mesangial matrix accumulation—mechanisms that protect against diabetic nephropathy [44]. Moreover, ISL has been proven to increase the synthesis of prostacyclin, which is a potent vasodilator. A reduced prostacyclin level has been noted in diabetic patients, which has resulted in atherosclerotic vascular complications [45]. Furthermore, ISL has also been shown to suppress the activation of α-glucosidase in type 2 diabetes [46].

### 3.4. Immunomodulatory Property of ISL

The therapeutic properties of ISL could rely on its ability to act against the influence of the toll-like receptor (TLR) pathway, which senses invading microbial organisms and initiates innate immunity. ISL inhibits NF-κB, which plays a major role in regulating the immune responses to infection. It also suppresses the activation of interferon regulatory factor 3 and interferon-inducible genes, suggesting that ISL can modulate the TRIF-dependent signaling pathways of TLR [47]. On the receptor level, ISL has been shown to reduce LPS-triggered TLR4 dimerization, leading to the inhibition of inducible nitric oxide synthase (iNOS) and COX-2 expression [48]. Moreover, ISL could target the molecule TANK-binding kinase 1, causing the downregulation of the TRIF-dependent signaling pathway [49]. Regulatory T cells (T_reg_) are essential for the control of immune responses and the prevention of autoimmune diseases. ISL has been found to increase regulatory T cell differentiation and enhance T_reg_ cell function in order to suppress effector T cell proliferation [50].

### 3.5. Anti-Angiogenic Property of ISL

ISL can inhibit vessel growth induced by the vascular endothelial growth factor (VEGF), and it can induce the expression of the pigment epithelium-derived factor that are inhibitory to angiogenesis. The application of topical ISL in in vivo experiments, which has led to the alleviation of corneal neovascularization has been reported [51]. Furthermore, ISL has been shown to disturb a variety of angiogenic activities, including invasion, migration, and tube formation in HUVEC in vitro assays [52]. In addition, ISL has been found to suppress the production of phorbol 12-myristate 13-acetate (PMA)-triggered matrix metalloproteinase (MMP), which contributes to angiogenesis [53].

### 3.6. Potential Role of ISL in Cancer Development

ISL exhibits direct growth inhibitory effects in various cancers, including cervical, breast, hepatoma, colon, prostate, etc. ISL has been shown to inhibit the growth of cervical cancer cells HeLa with increased apoptosis and ROS production [54]. Alternatively, ISL has markedly impeded the proliferation of both prostate cancer cell lines C4-2 and LNCaP by notably decreasing the level of ROS and the mitochondrial membrane potential without affecting normal epithelial cells such as intraepithelial carcinoma (IEC-6) [55]. Moreover, it shows significant anticancer activities in DU145 prostate cancer cells through the alteration of the cell cycle progression, invasion, and migration. [56,57]. ISL has also been proven to significantly inhibit the growth of tumor xenograft in mice, established from MDA-MB-231 breast cancer cells [58]. For adenoid cystic carcinoma (ACC), ISL can effectively suppress cancer cell proliferation, migration, and tube formation of human endothelial hybridoma (EAhy926) cells in vitro. However, the effect of the EAhy926 cells ceased when VEGF was present or added directly. ISL could also suppress tumor angiogenesis, specifically via the down-regulation of mTOR pathway-dependent VEGF production in ACC cells, correlating with the concurrent activation of c-Jun NH2-terminal kinase (JNK) and the inhibition of extracellular signal-regulated kinase (ERK) [59,60]. Our recent research has unveiled the fact that except for typical anticarcinogenic mechanisms such as proapoptotic activity and the promotion of phase-specific cell cycle arrest, ISL, together with another herbal flavonoid called formononetin, could act by inducing a novel protein called NSAID-activated gene-1 (NAG-1) through the mediation of its upstream regulator Egr-1 in colon cancer cells [61]. Despite the diversified anticancer potential of ISL in a panel of human cancer types [62], there has been no report on its activity in pancreatic cancer so far.

### 3.7. ISL Modulates Antioxidant Enzymes and Phase II Enzymes in Neuroprotection and Chemoprevention

ISL has shown good radical scavenging activities against the superoxide anion radical, hydrogen peroxide, and hydroxyl free radicals. The ROS-quenching power of ISL involves the maintenance of the enzymatic antioxidant defense mechanisms mediated via superoxide dismutase (SOD), catalase (CAT), and glutathione peroxidase (GSH-Px) [63]. Through its radical-scavenging action, ISL exerts a neuroprotective effect by targeting and reverting mitochondrial membrane potential collapse that can help to rectify mitochondrial dysfunction due to oxidative stress. Hydrogen peroxide is rapidly generated from highly active intracellular superoxide, which can be rapidly converted by mitochondrial SOD2. A high level of hydrogen peroxide will facilitate further conversion into the more detrimental hydroxyl radicals, which can be degraded by CAT and GSH-Px. ISL helps to alleviate oxidative stress through the maintenance of these endogenous antioxidant defense systems.

The induction of Phase II metabolic enzymes can protect cells against the toxicity brought forth by ROS. ISL has been shown to induce NAD(P)H:quinone oxidoreductase 1 (NQO1), also named quinone reductase (QR), which is a Phase II enzyme that deactivates electrophiles and radicals [64]. The reduction of electrophilic quinones by QR is an important detoxification mechanism in the body that converts quinones into hydroquinones in order to reduce oxidative cycling. Many studies have shown that the induction of QR correlates with protection against chemical carcinogenesis in animal studies [65]. The induction of the Phase II enzyme QR by ISL selectively activates the antioxidant response element (ARE) through Keap1-Nrf2 signaling, which has resulted in tumor latency. Nonetheless, it is remarkable that ISL does not possess Phase I enzyme-inducing and cytochrome P450-activating properties, which makes it a “monofunctional” metabolic enzyme inducer.

## 4. ISL and Pancreatic Cancer

### 4.1. ISL Promotes Apoptosis in Cancer Cells by Homeostatic Regulation of ROS

The basal level of autophagy is elevated in most human PDAC, which is accompanied by the accumulation of ROS during the development and progression of pancreatic cancer [66,67]. In turn, the loss of autophagy can cause the accumulation of damaged mitochondria and facilitate the oxidative protein folding machinery, which further promotes ROS production. Apoptosis can be triggered by endoplasmic reticulum (ER) stress due to the buildup of these misfolded proteins in the ER. On the one hand, prolonged or severe ER stress promotes several pro-apoptotic factors that result in apoptosis, while on the other hand, it also activates a set of signaling pathways called unfolded protein response (UPR) to prevent apoptosis. The accumulation of misfolded proteins in the ER to a level that exceeds the ER chaperone folding capacity is a major factor that exacerbates protein aggregation, a phenomenon commonly occurring in neurodegenerative diseases. Alternatively, the perturbation of ER homeostasis also plays critical roles in tumorigenesis, whereas the therapeutic modulation of ER chaperones and/or UPR components presents potential anti-tumor treatments [68]. Reducing ROS production by antioxidants or chemical chaperones has provided an effective strategy to prevent protein misfolding and aggregation. We have previously demonstrated that a phytochemical cryptotanshinone restored apoptosis in colon cancer cells by the attenuation of UPR [69].

Although ISL is a natural antioxidant, we have shown that ISL increased ROS levels through the inhibition of autophagy in pancreatic cancer cells. It was reported that autophagy in PDAC facilitates tumor growth by preventing the accumulation of genotoxic levels of ROS as well as sustaining oxidative phosphorylation by providing bioenergetic intermediates. However, it is worth mentioning that ROS also exhibits paradoxical effects on tumor development, as both the induction and inhibition of ROS could promote cell death in cancer cells, including that in pancreatic cancer by disrupting the redox balance [70]. ROS promotes the initiation of carcinogenesis as well as the malignant transformation of cells at mild-to-moderate elevated levels, while excessive ROS evokes irreversible oxidative damage and triggers programmed cell death, dramatically causing permanent damage in cancer cells. It has been reported that pancreatic cancer cells with low levels of ROS are more resistant to chemotherapy [71]; therefore in established pancreatic cancers, simply increasing the ROS levels could kill the cancer cells. This is the main mode of action of many conventional chemotherapies, which we observed to have manifested in the proapoptotic effect of ISL in pancreatic cancer. A similar phenomenon of ISL-induced apoptosis can be found in HeLa and ovarian carcinoma SKOV-3 cells by increasing the intracellular ROS levels [72].

Most of the orthodox anticancer agents, including 5-FU and gemcitabine, could kill cancer cells by promoting apoptosis through the induction of ROS generation [73]. However, prolonged treatment with the same drug reduces the ROS level in cancer cells and eventually leads to drug resistance. ISL could synergistically inhibit the growth of pancreatic cancer with 5-FU, where similar synergy was not found when co-administered with gemcitabine (data submitted for publication). This may explain why gemcitabine can induce the accumulation of ROS while increasing the capacity of antioxidant programs, but in turn ending up with dropped levels of ROS that leads to intrinsic resistance to treatment [74]. Hence, the most important point to know when establishing anticancer strategies through the modulation of ROS in pancreatic cancer cells is to confirm the threshold level of ROS and the ratio of ROS to antioxidants in the system after chemotherapeutic drug treatment.

### 4.2. Antioxidant Role of Autophagy

Antioxidant response and autophagy are mechanisms that are simultaneously induced by oxidative stress conditions in order to reduce ROS levels in the body and attenuate oxidative damage to biomolecules and organelles being orchestrated in a homeostatic approach. Autophagy is a major degradation pathway in the cell that works closely with the ubiquitin-proteasome system to remove damaged organelles and aberrant macromolecules for the prevention of cell injury and cellular dysfunction. It is an essential body process for the regulation of redox balance during stressful conditions. Treatment with an autophagy-enhancing agent could help to reduce oxidative stress and alleviate inflammation [75]. ROS is an early inducer of autophagy upon nutrient deprivation; thus, treatment with antioxidants could partially or completely revert the process. However, a redox-independent relationship between autophagy and antioxidant response also exists through the Nrf2 pathway, which provides a new insight into the interconnection between autophagy and oxidative stress [76]. Excessive ROS is implicated in many diseases, including cancer, neurodegeneration, and aging, while low levels of ROS is a cellular signal that can induce autophagy and the antioxidant pathway in the body under physiological and pathological conditions. Conventional antioxidants such as the autophagy activator alone may not be ideal for the treatment of diseases characterized by both oxidative stress and autophagy dysfunction. Alternatively, the use of natural compounds with the dual targeting of antioxidant and autophagy could be a potential therapeutic direction in such cases [77].

Classical antioxidant therapy using ROS scavengers that acts by alleviating the cellular damage caused by oxidative stress may be insufficient in treating Alzheimer’s disease or Parkinson’s disease. Autophagy has been proposed to be an essential cellular antioxidant process that can be used as an alternative approach to compensate for these limitations [78]. It was found that some classes of antioxidants such as vitamin E and NAC, in addition to their ROS scavenging ability, will impair basal and induced autophagy in a series of clinical applications even beyond neurodegenerative diseases, including acetaminophen poisoning and malignant diseases [79]. In recent years, more evidence has suggested that in redox homeostasis, ROS–antioxidant interactions can act as a metabolic interface for signals derived from glycolytic/oxidative metabolism and the tumor microenvironment, while autophagy plays a central role during metabolic reprogramming that could provide a new therapeutic opportunity [80].

The redox signaling in autophagy involves crosstalk between oxidative stress and the autophagic machinery. As mentioned above, antioxidant treatment prevents autophagy, suggesting that redox imbalance has a pivotal role in driving the process. Such a rapid induction of autophagy upon ROS production from the mitochondria requires the mediation by redox-sensitive proteins such as AMPK. AMPK is an AMP-sensitive protein kinase that serves as an energy stress sensor in cells [81]. When AMPK is activated by reducing glucose consumption, which reduces cellular ATP, it will increase mitochondrial and oxidative metabolism gene expression by regulating transcriptional events [82]. The loss of AMPK activity has been observed in pancreatic cancer and liver cancer [83,84], and it is associated with the reprogramming of tumor cell metabolism associated with cell growth and proliferation [85]. It is also known that upon exposure to hydrogen peroxide, AMPK will be activated through the S-glutathionylation of reactive cysteines with the formation of a mixed disulphide, shifting the intracellular redox environment towards more oxidizing conditions. This signifies the importance of thiol homeostasis in autophagy induction [76].

### 4.3. ISL and Autophagy in PDAC Regulation

**Figure 1:**
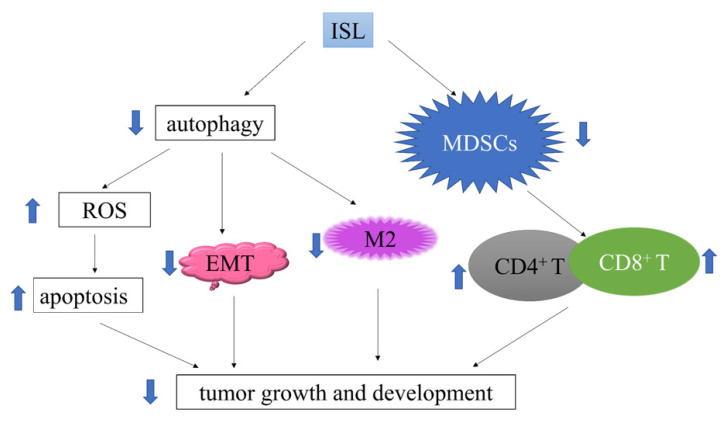
ISL inhibits pancreatic ductal adenocarcinoma cell growth by the blockade of autophagy and the regulation of the tumor microenvironment, leading to the increased activation of reactive oxygen species and the promotion of apoptosis together with the immunomodulation of tumor immunity.

In the context of pancreatic cancer, autophagy is believed to have played a prominent role in tumor maintenance and chemoresistance, suggesting its potential as a therapeutic target. It is worth emphasizing that autophagy is not a static status but a dynamic process [86], known as autophagy flux, which includes the formation of autophagosome and autolysosome, the degradation of delivered cargos, and the utilization of degradation products. In our study, we have determined the promotional effect of ISL on the expression of p62/SQSTM1, a selective substrate of autophagy (data submitted for publication). During the autophagy process, p62/SQSTM1 is usually incorporated into the completed autophagosome and is degraded in autolysosomes, which renders it with an index of autophagic degradation under certain circumstances. As a matter of fact, the downregulation of p62/SQSTM1 may correlate with autophagy activation [87]. Thus, the monitoring of LC3II levels in the absence and presence of autophagy inhibitors such as CQ or bafilomycin A1 will become essential in differentiating whether p62 downregulation really represents activated autophagy or is due to a block in fusion or degradation instead, especially in the case when LC3II is increased. In general, if autophagy occurs, the level of LC3II in a combined drug and late-stage inhibitor treatment group should be higher than that with the inhibitor alone [88]. If the treatment by one drug, and not by a combined drug and late-stage autophagy inhibitor, produces increased LC3II levels as compared to the use of an inhibitor alone, this may indicate that drug treatment in fact induced the complete or partial blockade of the autophagic flux [89]. This has been proven to be the case of ISL in our study, which is the first one to report that ISL could block autophagy in pancreatic cancer cells. Based on these findings, we may consider designating ISL as a natural autophagy inhibitor that can be used to replace conventional agents such as CQ, which may cause serious systemic side effects. A schematic plot of the mechanisms of action of ISL in PDAC, shown in Figure 1, is based on our current investigations, including findings that are beyond the scope of this review.

## 5. Insight from the ISL-Calysosin Isoflavonoid Biosynthetic Pathway

**Figure 2:**
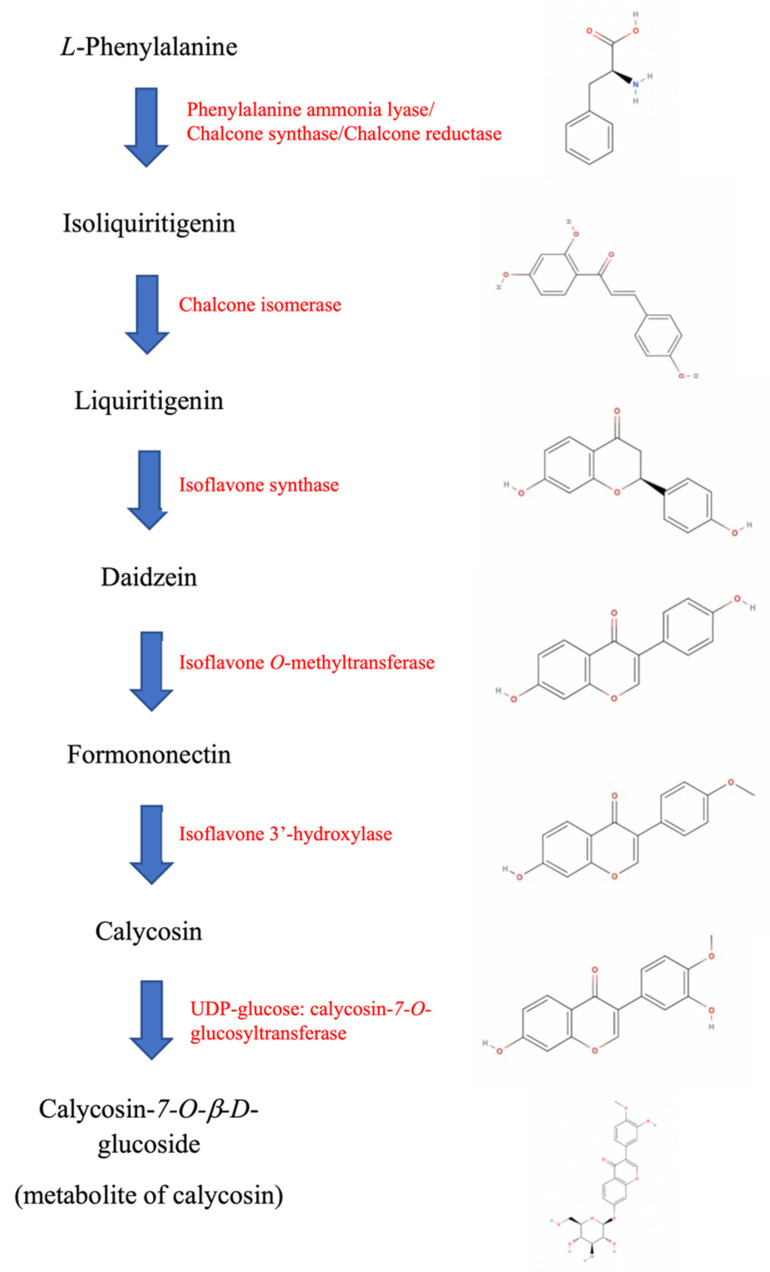
Biosynthetic pathway of herbal isoflavonoids—the ISL-calycosin cascade.

The isoflavone compounds of roots are often related to the bioactivity of the plants or herbs [90]. According to the biosynthetic pathway of herbal isoflavonoids, ISL is synthesized from L-phenylpropranoid via the isoflavonoid branch of phenypropanoid metabolism [91]. Through a series of enzymes, including phenylalanine ammonia lyase, chalcone synthase, chalcone reductase, chalcone isomerase, isoflavone synthase, isoflavone *O*-methyltransferase, and isoflavone 3′-hydroxylase, ISL will be converted into another important herbal isoflavonoid known as calycosin (Figure 2). Calycosin can also be found in licorice but is mainly obtained from a TCM lead herb called *Astragalus membranaceus* (“Chinese *Huangqi*”). In China, *Huangqi* decoctions have been included as a good health supplement that supports athlete’s humoral and cellular immunities after high-intensity training [92,93]. Consuming *Huangqi* would be beneficial as immunomodulators to strengthen the body in recovery and for the prevention of sickness. Compared to its medical functions, *Huangqi* is also treated as a flavoring agent for making tea and fish or chicken stew [94]. We have been studying *Astragalus Radix* and, in particular, its total saponins for more than a decade. A summary of the bioactivities of this herb and the underlying mechanisms of action of its active medicinal components have been presented in a review [95].

**Figure 3:**
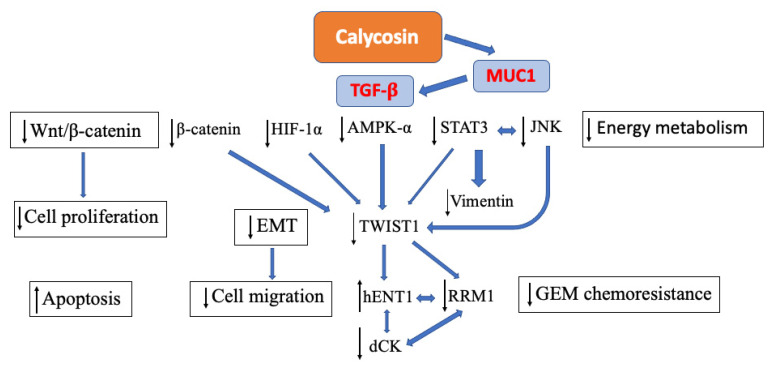
Calycosin inhibits pancreatic ductal adenocarcinoma progression by the promotion of apoptosis, the inhibition of cell proliferation and cell migratory activities, the facilitation of metabolic regulation, and the restoration of chemosensitivity.

In our recent investigation, we envisaged that calycosin inhibited the growth of pancreatic cancer cells by inducing p21-induced cell cycle arrest and caspase-dependent apoptosis. Alternatively, it also promoted MIA PaCa-2 PDAC cell migration by eliciting EMT and MMP activation. An in vivo study further confirmed the pro-invasive and angiogenic drive of calycosin and the subsequent EMT promotion in pancreatic tumors. These events appear to be associated with the increased expression of TGF-β1, which may explain the paradoxical drug actions since TGF-β has been implicated in playing dual roles as both the tumor suppressor and the tumor promoter in pancreatic cancer development [96]. Despite the biosynthetic relationship between ISL and calycosin, we also confirmed the close interactions between PDAC and both compounds as key molecules obtained from *Glycyrrhiza radix* and *Astragalus membranaceus* via a heatmap of network pharmacology analysis. From the mechanistic point of view, calycosin effectively inhibited pancreatic cancer cell migration through the inhibition of the epithelial-mesenchymal transition (EMT) and the promotion of early-stage apoptosis, as well as facilitated metabolic modulation in PDAC through the regulation of AMPKα signaling. Moreover, calycosin also restores the chemosensitivity in pancreatic cancer cells by the regulation of RRM1 signaling, a key mediator that causes gemcitabine chemoresistance. (Figure 3). The unique pro-metastatic potential of calycosin could be alleviated through the genetic knockdown of the TGF-β regulator MUC1 [97]. These phenomena have raised the speculation as to whether the anticancer effects and metabolic regulation induced by ISL are possibly not unique, and that they may also occur in other herbal isoflavonoids from the same biosynthetic cascade, with some differentiation in their respective actions.

## 6. Conclusions and Future Perspectives

Despite the fact that the prognoses of other cancers continue to improve over the years, the incidence of pancreatic cancer shows an increasing trend. It has been predicted to become the second leading cause of cancer death in Western countries by 2030, surpassing hepatocellular, colon, lung, and prostate cancers. In one study, about 75% of the patients suffering from PDAC died within one year of initial diagnosis [98]. Furthermore, the survival benefit has so far not been substantially improved by gemcitabine-based combination therapies due to the profound chemoresistance and serious systemic toxicity being brought forth by the drug [99]. The poor efficacy of combination therapy, which uses gemcitabine with other chemotherapeutic drugs such as erlotinib, nab-paclitaxel, and oxaliplatin, could be due to its cross-resistance to multiple drugs [100]. Hence, the search for a potential neoadjuvant agent that is capable of alleviating gemcitabine chemoresistance would certainly be beneficial to patients who are out of treatment options.

In order to improve chemotherapy for PDAC, the modulation of the tumor microenvironment and the stromal components are of great significance, especially regarding their contribution to chemoresistance [101]. A growing body of literature has suggested that the activation of autophagy could facilitate chemoresistance in different cancer cell lines [102]. Autophagy was determined to have the cytoprotective effect against anticancer drugs, such as 5-FU and gemcitabine in pancreatic cancer cells [103]. The impairment of tumor metabolism induced by the inhibition of autophagy may change the in situ anti-tumor immune responses [104]. Thus, the combination of conventional chemotherapy with a neoadjuvant capable of autophagy inhibition may be a promising therapeutic strategy for pancreatic cancer [105]. There is great potential that isoflavonoids from a herbal source could contribute to addressing the problem of chemoresistance and may improve the survival rate and quality of life of pancreatic cancer patients.

One of the unanswered conceptual questions on how autophagy could be targeted concerns the level of the pathway at which inhibition would be most optimal. The inhibition of earlier phases of the process, such as those involved in autophagosome biogenesis, would allow for the buildup of toxic protein aggregates and damaged mitochondria that would no longer be encompassed by the autophagosome and allow the tumor cells to be continuously exposed to these toxic insults [106]. However, the inhibition of the later steps, such as the lysosome, may have the advantage of inhibiting other metabolic scavenging pathways such as macropinocytosis, which has also been shown to be critical for tumor metabolism and growth [107]. In many cases, autophagy-upregulating agents mainly induce basal or early-phase autophagy, which could be alleviated by antioxidant drugs concurrently taken via classic autophagy regulatory pathways [79]. This suggests that some level of ROS production or redox signaling is indeed required for the effective regulation of autophagy due to the close and sometimes paradoxical relationship between the two entities. If an anticancer drug mainly acts through classical approaches such as antioxidation and mechanisms confined to the induction of programmed cell death and growth inhibition, the impact may not be sufficient to eradicate tumor cells. Since ISL possesses strong antioxidant properties and has also exhibited superb action in modulating both autophagy and redox regulation, it is regarded as a good choice for further development as a potential neoadjuvant in the chemotherapy of malignant neoplasms such as pancreatic cancer.

## Figures and Tables

**Table 1:** Names and Classification of *Glycyrrhiza Radix*.

Pharmaceutical Name:	*Glycyrrhiza Radix*
English name:	Licorice root
Common Names:	Licorice root, licorice, liquorice, sweet root, Gan Cao (Chinese licorice)
Family:	Fabaceae
Common species in Latin:	*Glycyrrhiza uralensis FISHCH*. (*Gan Cao*, Chinese licorice) *Glycyrrhiza inflate BAT.* (*Zhang Guo Gan Cao*) *Glycyrrhiza glabra* L. (*Guang Guo Gan Cao*) *Glycyrrhiza glabra* (European licorice) *Glycyrrhiza lepidota* (American licorice)

**Table 2:** The 2D and 3D structures of major isoflavonoids in licorice (derived from the MolView software).

Name of Isoflavonoid	Molecular Structures	
Glycyrrhizin	* 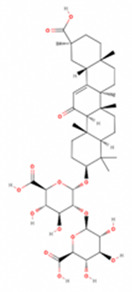 *	* 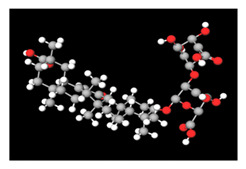 *
18α-Glycyrrhetinic acid	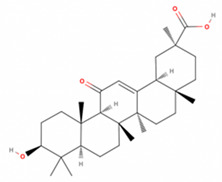	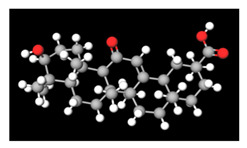
18β-Glycyrrhetinic acid	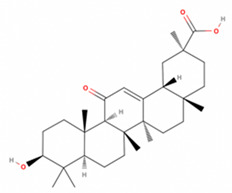	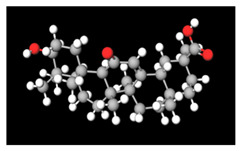
Licochalcone A	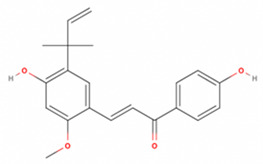	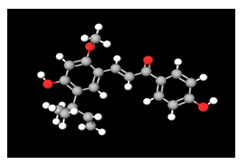
Licochalcone E	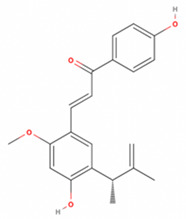	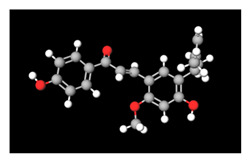
Glabridin	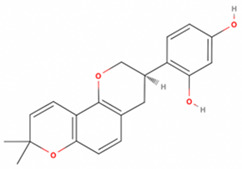	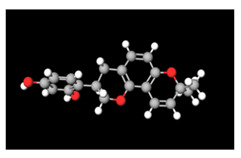
Liquiritigenin	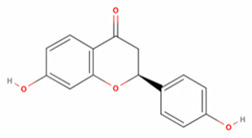	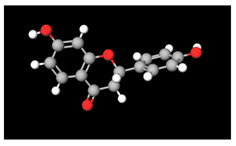
Isoliquiritigenin	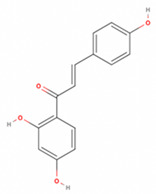	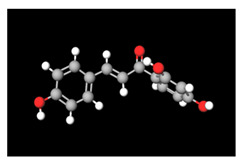

## Data Availability

The data are contained within the article.
